# Bioactive compounds, antioxidant activity and fruit quality evaluation of eleven blood orange cultivars

**DOI:** 10.1002/jsfa.11636

**Published:** 2021-11-19

**Authors:** Pilar Legua, Giulia Modica, Ignacio Porras, Agustín Conesa, Alberto Continella

**Affiliations:** ^1^ Plant Science and Microbiology Department Miguel Hernández University Alicante Spain; ^2^ Department of Agriculture, Food and Environment University of Catania Catania Italy; ^3^ Department of Citriculture Instituto Murciano de Investigación y Desarrollo Agrario y Alimentario (IMIDA) Murcia Spain

**Keywords:** anthocyanins, antioxidant activity, blood orange, *Citrus sinensis*, phenols

## Abstract

**BACKGROUND:**

Blood oranges are grown increasingly in Europe for fresh consumption because of their special taste and excellent nutraceutical properties that confer the status of a functional food. The health benefits are associated with the range of additional bioactive compounds that they contain with respect to blonde oranges.

**RESULTS:**

We analysed the physicochemical properties and the levels of organic acids, sugars and antioxidants in 11 blood orange cultivars representing the most representative cultivars of blood oranges widespread in the Mediterranean basin. In particular, we examined the levels of phenols, flavonoids and anthocyanins present in these cultivars at harvest maturity. The physicochemical, antioxidant and colour properties differ significantly among these cultivars. The deepest red peel and juice was found in Sanguinelli, followed by Tarocco Rosso and Moro. High‐performance liquid chromatography with refractive index detector analysis revealed sucrose as the main sugar in all these cultivars, followed by fructose and glucose. Citric acid was the dominant organic acid, followed by malic acid and ascorbic acid. Moro showed the greatest levels of antioxidant activity. Regarding the phenolic composition, we found *p*‐coumaric acid to be the main hydroxycinnamic acid in all cultivars, with maximum amounts in Moro and Sanguinelli. The highest amounts of cyanidin‐3‐*O*‐glucoside and cyanidin‐3‐(6′′‐malonyl)‐glucoside were found in Moro, for which the juice was of the deepest red colour.

**CONCLUSION:**

The phenolic composition and antioxidant activity of the 11 cultivars was assessed. The results showed that Moro was the cultivar with the highest content of polyphenols and levels of antioxidant activity, followed by Sanguinelli. © 2021 The Authors. *Journal of The Science of Food and Agriculture* published by John Wiley & Sons Ltd on behalf of Society of Chemical Industry.

## INTRODUCTION

Sweet oranges (*Citrus sinensis* L. Osbeck) are one of the most important citrus fruits. Total world production of sweet oranges is around 79 million tons.^1^ The two major groups of sweet oranges are blonde oranges, which are widespread in almost all citrus‐producing countries, and blood oranges, which are grown in relatively few regions and where the climate favours the synthesis of the red pigment. The red colour of blood oranges is a result of the high levels of the water‐soluble pigment, anthocyanin, which belongs to the larger family of flavonoids. The amounts of these pigments, both in the peel and in the flesh, depend on a range of factors, including cultivar, rootstock, maturity, region of cultivation and environment.^2^, ^3^ Of the latter, the thermal excursions between night and day are considered to be a crucial factor in stimulating anthocyanin synthesis.^4^, ^5^ For these reasons, the pigmented cultivars, the so‐called blood oranges, are cultivated mainly in Italy and, especially, in a Sicilian production district of almost 40 000 ha characterised by a Protected Geographical Indication (GPI). These fruits are mostly consumed fresh.^6^ The most important Italian blood orange cultivars are Tarocco and Moro. The first is characterised by a number of clones with different maturation periods. It is consumed as a fresh fruit and favoured for its easy peelability and high levels of sugar/acid ratio. Moro is the most deep red in colour and its juice offers some anti‐inflammatory effects as a result of the high levels of certain bioactive compounds^7^ including flavonoids that are important in the human diet.^8^

In Spain, red oranges are cultivated on 952 ha and represent approximately 0.7% of total sweet orange production there, whereas more than 72.3% are represented by early and late navels.^9^ The most common blood orange is cv. Sanguinelli, a spontaneous mutation of cv. Doble Fina. Although Sanguinelli and Moro are the most colourful, a number of pigmented cultivars have been grown in Spain, including cv. Doble Fina and its natural mutations, cv. Entrefina and the very similar cv. Murtera. Both are characterised by a lower juice pigmentation but are very rich in phenolic compounds.^10^ The cultivar Maltaise demi Sanguine is of unknown origin and it is grown extensively in Tunisia and, to a lesser extent, in Morocco.^11^

Recently, increased interest has been shown in blood oranges as a good source of natural antioxidants and bioactive compounds such as polyphenols (flavonoids, anthocyanins, hydroxycinnamic acids) and higher amounts of ascorbic acid to respect to blonde oranges.^12^, ^13^ Antioxidants play important roles in the human diet.^14^ In particular, epidemiological studies show their antioxidant and anti‐inflammatory activities have beneficial effects on human health, lowering the risk of cardiovascular diseases, diabetes and cancers.^14^, ^15^

In this context, the present study characterises 11 cultivars of blood orange, grown under the same environmental conditions, with respect to their contents of phytochemical compounds and bioactive properties.

## MATERIALS AND METHODS

### Cultivars and fruit sampling

Fruits of the blood orange cultivars were collected from the principal citrus germplasm bank of Spain (latitude 39°35′22.6′′N × 0°23′41.0′′W, Coord UTM, ETRS 89 Huso 30, X: 723678 Y: 4385379) located at the Instituto Valenciano de Investigaciones Agrarias (IVIA) in Valencia, Spain. The study included the fruits of 11 cultivars of blood orange: Doble Fina, Entrefina, Maltaise Blonde, Maltaise demi sanguine, Moro, Murtera, Sanguinelli, Tarocco Comune, Tarocco Messina, Tarocco Rosso and Washington Sanguine. All cultivars were grafted on the same hybrid rootstock Carrizo citrange.

For each cultivar, three replicates samples, each consisting of 10 fruits (*n* = 30) were harvested over two seasons (2017 and 2018). The fruit were hand‐harvested at physiological maturity, aiming to ensure best flavour and colour, and immediately transported under well‐ventilated conditions to the laboratory.

### Physical and chemical determinations

Once in the laboratory, 30 oranges per cultivar were selected for analytical determination. Fruits of three subsamples per cultivar (each of 10 fruits) were cut in half and carefully hand‐squeezed in a commercial juicer. The fresh juices were centrifuged at 13 000 × *g* for 20 min (model 3‐18K; Sigma, Darmstadt, Germany).

Fruit weights were determined with a digital balance (accuracy of 0.01 g) (model BL‐600; Sartorius, Göttingen, Germany). Fruit size (equatorial diameters and length) and pulp thickness were measured with an electronic digital slide gauge (accuracy of 0.01 mm) (model CD‐15 DC; Mitutoyo, Kawasaki, Japan).

Total soluble solids (TSS) contents were measured with a digital refractometer Atago N1 (0.2 °Brix) (model N‐1; Atago Co. Ltd, Tokyo, Japan) at 20 °C. The titratable acidity (TA) was determined using an automatic titration device (877 Titrino plus, Metrohm, Herisau, Switzerland) with 0.1 n NaOH up to pH 8.1, and results expressed as g citric acid L^−1^ because this is the dominant organic acid in oranges. Once the TSS and TA contents had been assessed, the maturity index (MI) was calculated as the TSS/TA ratio. Results are shown as the mean ± SE.

Colour determinations were made of both the rind (*n* = 120) and juice (*n* = 12) according to the Commission Internationale de lʼ Éclairage (CIE) and expressed as L*, a*, b*: L* (brightness or lightness; 0 = black, 100 = white), a* (−a* = greenness, +a* = redness) and b* (−b* = blueness, +b* = yellowness). These values were then used to calculate Hue angle degree [H°* = arctang (b*/a*)], where 0° = red–purple; 90° = yellow, 180° = bluish–green and 270° = blue and chroma [C* = (a*2 + b*2)]1/2, indicate of the colour intensity or saturation. As suggested in previous studies,^16^ hue angle (*H*°*) and chroma (C*) have been accepted as the more intuitively understandable colour variables. Colour index (CI) was calculated as: CI = 1000 a*/L*b*.^17^ Colour variables were measured using a Minolta C‐300 Chroma Meter (Minolta Corp., Osaka, Japan) coupled to a DP‐301 data processor (Minolta Corp.).

### Determination of sugars and organic acids

Individual organic acids and sugars were also determined using three juice samples for each cultivar as described in previous research.^18^ Briefly, 1 mL of the centrifuged juice was passed through a 0.45‐μm filter (Millipore, Burlington, MA, USA) and injected into a HP 1100 series high‐performance liquid chromatography (HPLC) system (Hewlett‐Packard, Wilmington, DE, USA). The elution system consisted of 0.1% phosphoric acid with a flow rate of 0.5 mL min^−1^. Organic acids were separated on a Supelcogel TM C—610H column (30 cm × 7.8 mm inner diameter; Supelco, Inc., Bellefonte, PA, USA) and Supelguard column (5 cm × 4.6 mm; Supelco, Inc.), and detected using a diode‐array detector set at 210 nm. For the sugar analyses, the same HPLC equipment, elution system, flow rate and columns were used. The detection of sugars was carried out using a refractive index detector (HP 1100 series, G1362A). Standard curves for pure standards of organic acids (oxalic, citric, malic, quinic and ascorbic acids) and for sugars (glucose, fructose and sucrose) (Sigma) were used for quantification. The results for both organic acids and sugars are expressed as concentrations g kg^−1^ fresh weight (FW). Sugars and organic acids were determined in triplicate.

### Total polyphenol content (TPC) and total antioxidant activity (TAA)

TPC was quantified using Folin–Ciocalteu reagent.^19^ Briefly, for each sample, 2 g of flesh tissue was homogenised in 5 mL of MeOH/water (80:20, v/v) + 2 mm NaF and centrifuged at 13 000 × *g* for 20 min. Absorption was measured at 760 nm using a spectrophotometer (ThermoSpectronic Heγios, Cambridge, UK). The results (mean ± SD) are expressed as mg of gallic acid 100 g^−1^ FW equivalent.

Total antioxidant activity was quantified as described previously.^20^ This procedure allows determination of both the hydrophilic and lipophilic TAA in the same extraction. Briefly, for each subsample, 5 g of flesh tissue was homogenised in 5 mL of 50 mm phosphate buffer (pH 7.8) and 3 mL of ethyl acetate, then centrifuged at 10 000 × *g* for 15 min at 4 °C. The upper fraction was used for TAA as a result of lipophilic compounds (L‐TAA) and the lower one for TAA as a result of hydrophilic compounds (H‐TAA). In both cases, TAA was determined in triplicate for each extract using an enzyme system composed of the chromophore 2,2′‐azino‐bis‐(3‐ethylbenzothiazoline‐6‐sulfonic acid) diammonium salt (ABTS), the horseradish peroxidase enzyme and its oxidant substrate (hydrogen peroxide), in which ABTS^•+^ radicals are generated and monitored at 730 nm. The decrease in absorbance after adding the extract was proportional to the TAA of the sample. A calibration curve was obtained using Trolox [(*R*)‐(+)‐6‐hydroxy‐2,5,7,8‐tetramethyl‐croman‐2‐carboxylic acid] (0–20 nmol) from Sigma and the results (mean ± SD) are expressed as mg of Trolox equivalent 100 g^−1^ FW.

### HPLC‐diode array detection‐electrospray ionization‐mass spectrometry (DAD‐ESI‐MS^n^ ) analyses: identification and quantification of phenolic compounds

Three samples of each of the 11 cultivars (*n* = 33) were frozen in liquid nitrogen, to be later freeze‐dried in an Alpha 2–4 freeze drier (Christ Alpha 2–4; Braun Biotech, Osterode am Harz, Germany) for 24 h at a pressure reduction of 0.220 mbar. The temperature in the drying chamber was −25 °C, whereas the heating plate reached 15 °C. Samples of 50 mg of freeze‐dried powder were mixed with 2 mL of 80% aqueous methanol acidified with formic acid (1%), vortexed and sonicated for 1 min at room temperature. The resulting heterogeneous mixture was centrifuged at 900 × *g* for 4 min and the supernatant passed through a 0.45 μm PTFE filter (Waters Corp., Milford, MA, USA) prior to injection into the chromatograph system.

Chromatographic analyses were carried out on an series 1100 HPLC‐ESI‐DAD‐MS^n^ Ion Trap (Agilent, Waldbronn, Germany). This HPLC system with DAD detector series 1100 was coupled to a mass spectrometer equipped with an ion trap and an ESI interface). The capillary temperature and voltage for the ESI source were set at 350 °C and 3500 V, respectively. The collision‐induced fragmentation occurred inside the ion trap using helium as the collision gas and a collision energy of 50%. The mass range for the precursor ions (MS) and their subsequent fragmentations (MS–MS) was from 100 to *m*/*z* 1000 and the data were acquired in negative ionisation mode, where the deprotonation of the molecules was observed. A reverse‐phase Pursuit XRs 5 C18 column was used (250 × 4.6 mm inner diameter and particle size 5 μm) (Agilent). Water/formic acid (95:5, v/v) and acetonitrile were used as the mobile phases A and B, respectively, with a flow rate of 0.8 mL min^−1^. The gradient started with 5% of solvent B, reaching 60% solvent B at 37 min, and 98% at 40 min, which was maintained up to 2 min. The injection volume was 10 μL. Chromatographic comparison with analytical standards, absorbance spectra and mass spectra, using MS^n^ data (not shown) were used to identify compounds. Flavanones and flavone C‐glucosides were monitored and quantified at 280 and 330 nm, whereas anthocyanins were monitored and quantified at 520 nm.

Cyanidin 3‐*O*‐glucoside, chlorogenic acid and *p*‐coumaric acid were purchased from Sigma, whereas naringin, hesperidin and vitexin were obtained from Extrasynthese (Lyon, France).

All analyses were carried out in triplicate. The results are reported as milligram of compound per liter (mg L^−1^) of juice.

### Statistical analysis

A basic descriptive statistical analysis was followed by an analysis of variance for mean comparisons. Fisherʼs least significant difference (LSD) procedure at a 95.0% confidence level was used to discriminate among the means (multiple range test). Principal component analysis (PCA) was performed. All statistical analyses were carried out using Statistica, version 6.0 (StatSoft, Inc., Tulsa, OK, USA).

## RESULTS

### Physicochemical properties

Table 1 lists the physicochemical properties, size, weight, soluble solids and titratable acidity. Maltaise demi Sanguine, Murtera and Sanguinelli showed significantly lower fruit weights (108, 113 and 113 g, respectively), whereas Tarocco Rosso (212 g), followed by Maltaise Blonde (197 g), Moro (196 g) and Washington Sanguine (189 g), had a higher mean fruit weight. Fruit equatorial diameters and height ranged between 58–74 mm and 64–75 mm, respectively.

**Table 1:** Physicochemical properties of blood oranges

	Fruit weight (g)	Equatorial diameter (mm)	Fruit height (mm)	Rind thickness (mm)	TSS (° Brix)	TA (g citric acid L^−1^)	MI (TSS/TA)
Doble Fina	158 ± 5.2 d	64.3 ± 2.1 d	73.2 ± 2.3 ab	3.6 ± 0.6 bc	12.45 ± 0.09 f	14.17 ± 0.22 a	8.78 ± 0.08 e
Entrefina	137 ± 6.8 e	65.5 ± 3.3 cd	62.0 ± 3.5 cd	4.4 ± 1.2 a	12.17 ± 0.06 g	13.94 ± 0.19 ab	8.73 ± 0.08 e
Maltaise Blonde	197 ± 20.5 b	70.0 ± 7.3 b	73.5 ± 6.5 ab	3.4 ± 1.3 bc	13.00 ± 0.10 c	11.99 ± 0.20 e	10.84 ± 0.15 b
Maltaise demi Sanguine	108 ± 4.6 f	59.8 ± 2.5 e	58.8 ± 2.6 d	4.0 ± 0.8 ab	12.63 ± 0.08 e	12.24 ± 0.25 de	10.32 ± 0.16 c
Moro	196 ± 11.7 b	70.5 ± 4.2 b	72.9 ± 3.9 ab	4.6 ± 0.6 a	12.80 ± 0.10 d	13.38 ± 0.29 c	9.57 ± 0.14 d
Murtera	113 ± 4.6 f	58.7 ± 2.4 e	60.8 ± 3.3 cd	3.2 ± 0.8 c	12.93 ± 0.06 cd	11.95 ± 0.17 e	10.82 ± 0.19 b
Sanguinelli	113 ± 3.3 f	58.0 ± 1.7 e	64.1 ± 3.7 c	3.6 ± 1.0 bc	12.03 ± 0.15 g	12.68 ± 0.52 d	9.50 ± 0.26 d
Tarocco Comune	138 ± 6.0 e	63.5 ± 2.8 d	60.5 ± 3.2 d	2.5 ± 0.6 d	15.03 ± 0.06 a	13.47 ± 0.54 bc	11.16 ± 0.43 ab
Tarocco Messina	174 ± 10.0 c	66.5 ± 3.8 cd	75.5 ± 4.4 a	3.2 ± 1.0 cd	11.33 ± 0.14 h	12.23 ± 0.12 de	9.26 ± 0.19 d
Tarocco Rosso	212 ± 12.6 a	74.5 ± 4.4 a	70.1 ± 2.4 b	3.5 ± 1.2 bc	12.13 ± 0.06 g	10.75 ± 0.33 f	11.30 ± 0.40 a
Washington Sanguine	189 ± 7.6 b	67.8 ± 2.7 bc	72.2 ± 7.0 ab	4.0 ± 0.6 ab	13.90 ± 0.10 b	13.60 ± 0.30 bc	10.22 ± 0.15 c

Data are the mean ± SD (*n* = 60). Values followed by the same lowercase letter, within the same column, are not significant different according to Fisherʼs LSD procedure at 95% confidence level.

The thickest rind was in Moro, Entrefina, Maltaise demi Sanguine and Washington Sanguine, whereas the thinnest was in Tarocco Comune and Tarocco Messina (2.5 and 3.2 mm, respectively).

Concerning the values of TSS and TA, fruits of Tarocco Comune showed the highest TSS content (15.03 °Brix), whereas Tarocco Messina fruits recorded the lowest TSS (11.33 °Brix). At the end of sampling, all the pigmented cultivars registered values of total acidity of between 11 and 14 g citric acid L^−1^.

### External rind and juice colour

Regarding rind and juice colour, all properties are summarised in Table 2. The *H*° values were lower in the peels of Sanguinelli, Tarocco Rosso, Murtera and Moro, whereas the highest were in the peels of Maltaise Blonde and Tarocco Messina. The high Citrus Colour Index (CCI) values observed in fruits of Sanguinelli confirmed the deep red colour. Maltaise blonde showed the lowest CCI value. The highest value of a* was found in Tarocco Rosso (13.83) and Tarocco Messina (13.67), and the lowest values were in Doble Fina (7.54) and Maltaise Blonde (7.42).

**Table 2:** Peel and juice colour of blood oranges

Paramete*r* ^a^	L*	a*	b*	C*	*H*°	CCI
Rind colour*						
Doble Fina	59.63 ± 2.08 b	27.89 ± 1.57 ef	39.72 ± 3.29 d	48.57 ± 3.01 ef	54.84 ± 2.46 bc	11.88 ± 1.46 de
Entrefina	59.39 ± 1.04 b	33.09 ± 1.21 a	39.92 ± 1.76 d	51.88 ± 1.26 cd	50.33 ± 1.91 de	14.01 ± 1.20 d
Maltaise Blonde	65.63 ± 2.20 a	31.33 ± 1.30 b	66.40 ± 2.83 a	73.45 ± 2.19 a	64.70 ± 1.73 a	7.24 ± 0.83 g
Maltaise demi Sanguine	58.77 ± 2.37 b	30.78 ± 1.30 bc	37.82 ± 4.78 de	48.87 ± 3.59 ef	50.62 ± 4.06 de	14.18 ± 2.71 cd
Moro	54.62 ± 2.17 c	30.51 ± 1.67 bc	34.90 ± 5.68 ef	46.47 ± 4.76 fg	49.49 ± 4.41 ef	16.48 ± 3.29 bc
Murtera	53.84 ± 2.98 c	29.57 ± 1.51 cd	32.78 ± 4.70 f	44.26 ± 3.64 g	47.70 ± 4.32 ef	17.24 ± 3.52 b
Sanguinelli	50.37 ± 3.35 d	27.34 ± 1.66 f	25.22 ± 5.52 b	37.34 ± 4.67 h	42.15 ± 5.13 g	22.63 ± 5.66 a
Tarocco Comune	59.54 ± 1.78 b	29.15 ± 1.13 de	46.02 ± 2.77 c	54.51 ± 2.04 c	57.59 ± 2.30 b	10.72 ± 1.24 ef
Tarocco Messina	63.70 ± 1.49 a	28.08 ± 1.87 ef	52.19 ± 2.19 b	59.31 ± 1.37 b	61.69 ± 2.45 a	8.50 ± 1.07 fg
Tarocco Rosso	54.49 ± 3.07 c	30.99 ± 1.86 bc	32.08 ± 4.52 f	44.75 ± 2.96 g	45.78 ± 5.09 fg	18.31 ± 4.45 b
Washington Sanguine	55.35 ± 4.04 c	29.68 ± 1.42 cd	39.68 ± 6.61 d	49.70 ± 5.50 de	52.79 ± 4.60 cd	14.07 ± 3.41 cd
Juice colour**						
Doble Fina	43.16 ± 3.08 d	7.54 ± 1.33 d	22.39 ± 2.86 d	23.69 ± 2.46 d	71.03 ± 4.88 a	8.21 ± 2.87 d
Entrefina	47.84 ± 1.62 a	9.21 ± 1.10 c	32.01 ± 2.02 a	33.31 ± 2.20 a	73.99 ± 1.17 a	6.02 ± 0.58 d
Maltaise Blonde	43.13 ± 0.97 cd	7.42 ± 1.08 d	25.11 ± 2.10 c	26.19 ± 2.30 c	73.59 ± 1.08 a	6.83 ± 0.44 d
Maltaise demi Sanguine	45.90 ± 1.32 b	8.60 ± 1.04 cd	28.68 ± 1.37 b	29.96 ± 1.38 b	73.30 ± 1.95 a	6.56 ± 0.92 d
Moro	30.38 ± 2.40 g	11.27 ± 3.81 b	9.22 ± 2.61 g	14.84 ± 3.55 e	39.95 ± 11.48 d	47.75 ± 37.27 a
Murtera	45.10 ± 2.91 bc	10.83 ± 1.28 b	28.70 ± 2.24 b	30.72 ± 1.97 b	69.23 ± 3.14 a	8.52 ± 1.80 d
Sanguinelli	34.30 ± 2.91 f	9.09 ± 2.45 c	11.92 ± 3.00 f	15.34 ± 1.86 e	52.13 ± 13.04 c	26.04 ± 15.81 b
Tarocco Comune	49.29 ± 3.21 a	11.69 ± 1.68 b	32.17 ± 4.19 a	34.26 ± 4.25 a	69.95 ± 2.61 a	7.50 ± 1.55 d
Tarocco Messina	41.87 ± 2.81 de	13.67 ± 1.70 a	26.01 ± 3.26 c	29.50 ± 2.46 b	61.96 ± 5.49 b	13.14 ± 4.34 cd
Tarocco Rosso	36.41 ± 0.93 f	13.83 ± 1.17 a	17.22 ± 1.21 e	22.11 ± 1.10 d	51.23 ± 3.32 c	22.23 ± 3.23 bc
Washington Sanguine	40.89 ± 1.61 e	8.22 ± 1.42 cd	21.95 ± 1.37 d	23.47 ± 1.45 d	69.52 ± 3.31 a	9.22 ± 1.91 d

Data are the mean ± SD. *n = 120; **n = 12. Values followed by the same lowercase letter, within the same column, are not significant different according to Fisherʼs LSD procedure at 95.0% confidence level.

^a^ L*, lightness; a*, green/red coordinate; b*, blue/yellow coordinate; C*, chroma; *H*°*, hue angle; CI, colour index.

**Figure 1:**
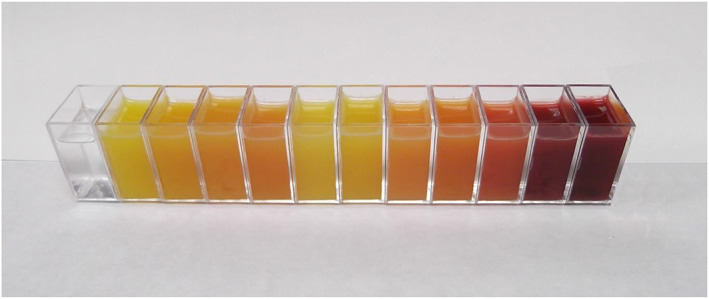
Juice of the eleven cultivars analysed: from left to right, distilled water, Entrefina, Murtera, Washington Sanguine, Doble Fina, Maltaise Blonde, Maltaise demi sanguine, Tarocco Comune, Tarocco Messina, Tarocco Rosso, Sanguinelli and Moro.

Based on *H*° and CCI, the juice of Moro was of deepest colour (Fig. 1), followed by Sanguinelli and Tarocco Rosso. Several cultivars showed pale red juice with values not significantly different from one another (Doble Fina, Entrefina, Maltaise Blonde, Maltaise demi Sanguine, Murtera, Washington Sanguine and Tarocco Comune).

### Organic acids and sugars

Marked differences were found in organic acid composition (Table 3), in which citric acid was the main organic acid, followed by malic and ascorbic acids. Doble Fina and Sanguinelli showed the highest concentrations of citric acid (20.5 and 18.1 g kg^−1^, respectively), Maltaise demi Sanguine (9.6 g kg^−1^) the lowest. However, these were not significantly different in Murtera (17.5 g kg^−1^), Moro (17.3 g kg^−1^), Washington Sanguine (17.2 g kg^−1^), Maltaise Blonde (16.7 g kg^−1^) and the three Taroccoʼs clones (ranging between 16.6 and 16.9 g kg^−1^).

**Table 3:** Organic acids and sugars of blood orange juice (g kg^−1^ FW)

Parameter	Malic acid	Ascorbic acid	Citric acid	Fructose	Glucose	Sucrose
Doble Fina	2.01 ± 0.13 ef	0.35 ± 0.01 c	20.49 ± 0.48 a	30.66 ± 0.73 bc	20.40 ± 0.45 bc	50.60 ± 0.51 d
Entrefina	2.37 ± 0.24 b	0.49 ± 0.03 a	13.39 ± 0.29 bc	32.40 ± 1.90 ab	22.20 ± 1.60 a	53.10 ± 1.05 bcd
Maltaise Blonde	1.96 ± 0.03 f	0.36 ± 0.02 c	16.65 ± 0.072 ab	29.26 ± 0.92 cd	20.30 ± 0.36 bc	58.46 ± 0.95 a
Maltaise demi Sanguine	2.03 ± 0.04 def	0.38 ± 0.03 c	9.60 ± 0.80 c	32.60 ± 1.34 ab	22.56 ± 0.92 a	59.43 ± 1.35 a
Moro	2.25 ± 0.06 bc	0.37 ± 0.02 c	17.33 ± 0.41 ab	32.70 ± 0.85 ab	22.33 ± 0.60 a	51.83 ± 1.19 cd
Murtera	2.20 ± 0.02 bcde	0.43 ± 0.01 b	17.49 ± 0.33 ab	33.43 ± 1.43 a	22.43 ± 0.66 a	53.73 ± 1.07 bc
Sanguinelli	2.23 ± 0.19 bcd	0.50 ± 0.04 a	18.12 ± 0.31 a	33.46 ± 2.65 a	21.73 ± 1.69 ab	53.10 ± 1.66 bcd
Tarocco Comune	2.06 ± 0.07 cdef	0.40 ± 0.01 ab	16.64 ± 0.53 ab	34.26 ± 1.07 a	22.66 ± 0.57 a	54.46 ± 1.01 b
Tarocco Messina	2.01 ± 0.09 ef	0.38 ± 0.02 bc	16.89 ± 0.68 ab	22.20 ± 1.05 e	14.60 ± 0.30 d	51.36 ± 1.00 cd
Tarocco Rosso	2.72 ± 0.11 a	0.38 ± 0.02 bc	16.92 ± 0.162 bc	27.73 ± 0.47 d	18.26 ± 0.32 c	52.70 ± 0.30 bcd
Washington Sanguine	2.39 ± 0.15 b	0.35 ± 0.03 c	17.18 ± 0.77 ab	32.06 ± 1.97 ab	21.80 ± 1.40 ab	58.43 ± 3.62 a

Data are the mean ± SD (*n* = 6). Values followed by the same lowercase letter, within the same column, are not significant different according to Fisherʼs LSD procedure at 95% confidence level.

The vitamin C content, calculated as ascorbic acid, had the highest levels in Entrefina and Sanguinelli (0.49 and 0.50 g kg^−1^, respectively). There were no significant differences between ascorbic acid levels in Doble Fina, Maltaise Blonde, Maltaise demi Sanguine, Moro or Washington Sanguine.

Sucrose was the main sugar in all cultivars, followed by fructose and by glucose (Table 3). The highest concentrations of sucrose were found in Maltaise demi Sanguine, Maltaise Blonde and Washington Sanguine (59.4, 58.5 and 58.4 g kg^−1^, respectively). Tarocco Comune (34.3 g kg^−1^), Sanguinelli (33.5 g kg^−1^) and Murtera (33.4 g kg^−1^) showed the largest contents of fructose, whereas Tarocco Messina had the lowest fructose content (22.2 g kg^−1^). Entrefina, Maltaise Blonde, Maltaise demi Sanguine, Tarocco Comune and Moro had the highest levels of glucose, ranging from 22.6 to 21.7 g kg^−1^, whereas the lowest level was in Tarocco Messina (14.6 g kg^−1^).

### Polyphenols and total antioxidant activity

TPC and TAA of the 11 blood oranges were listed Table 4. The TPC ranged between 117.26 and 241.91 mg of gallic acid 100 g^−1^. Maximum phenolic contents were recorded in the juices of Tarocco Messina, Tarocco Rosso and Doble Fina (241.9, 236.34 and 222.02 mg gallic acid 100 g^−1^, respectively). Tarocco Comune showed the lowest values (117.26) together with Maltaise demi Sanguine and Maltaise Blonde (131.51 and 133.59, mg gallic acid 100 g^−1^, respectively).

**Table 4:** Polyphenols and TAA of blood orange juice

Parametera	TPC (mg gallic acid 100 g^−1^)	H‐TAA (mg Trolox 100 g^−1^)	L‐TAA (mg Trolox 100 g^−1^)
Doble Fina	222.02 ± 11.78 a	89.72 ± 5.92 bcde	1.35 ± 0.05 a
Entrefina	163.10 ± 24.21 cd	82.12 ± 2.41 cde	1.55 ± 0.18 a
Maltaise Blonde	133.59 ± 1.56 ef	75.22 ± 8.49 e	1.35 ± 0.20 a
Maltaise demi Sanguine	131.51 ± 12.90 ef	75.47 ± 1.11 de	1.41 ± 0.23 a
Moro	188.89 ± 8.80 bc	129.75 ± 23.79 a	1.43 ± 0.10 a
Murtera	192.58 ± 4.53 b	96.87 ± 11.98 bc	1.52 ± 0.28 a
Sanguinelli	157.64 ± 19.94 de	104.38 ± 5.43 b	1.52 ± 0.05 a
Tarocco Comune	117.26 ± 17.16 f	82.18 ± 4.59 cde	1.38 ± 0.13 a
Tarocco Messina	241.91 ± 19.45 a	85.08 ± 8.07 cde	1.55 ± 0.28 a
Tarocco Rosso	236.34 ± 25.30 a	91.29 ± 4.46 bcd	1.35 ± 0.13 a
Washington Sanguine	168.57 ± 4.30 bcd	84.43 ± 5.33 cde	1.29 ± 0.10 a

Data are the mean ± SD (*n* = 6). Values followed by the same lowercase letter, within the same column, are not significant different according to Fisherʼs LSD procedure at 95.0% confidence level.

TPC, total polyphenols content; H‐TAA, hydrophilic total antioxidant activity; L‐TAA, lipophilic total antioxidant activity.

Our records of hydrophilic antioxidant activity (H‐TAA) were significantly higher than those of lipophilic antioxidant activity (L‐TAA) (Table 4). Moro showed the highest level (129.75 mg Trolox 100 g^−1^). No significant differences were found between levels in Doble Fina, Tarocco Messina, Washington Sanguine, Tarocco Comune, Entrefina, Maltaise demi Sanguine and Maltaise Blonde, with the latter two showing the lowest antioxidant activities (75.47 and 75.22 mg Trolox 100 g^−1^, respectively). The results of lipophilic TAA among the 11 cultivars were not significantly different.

### Total anthocyanins, hydroxycinnamic acids and phenolic composition

**Figure 2:**
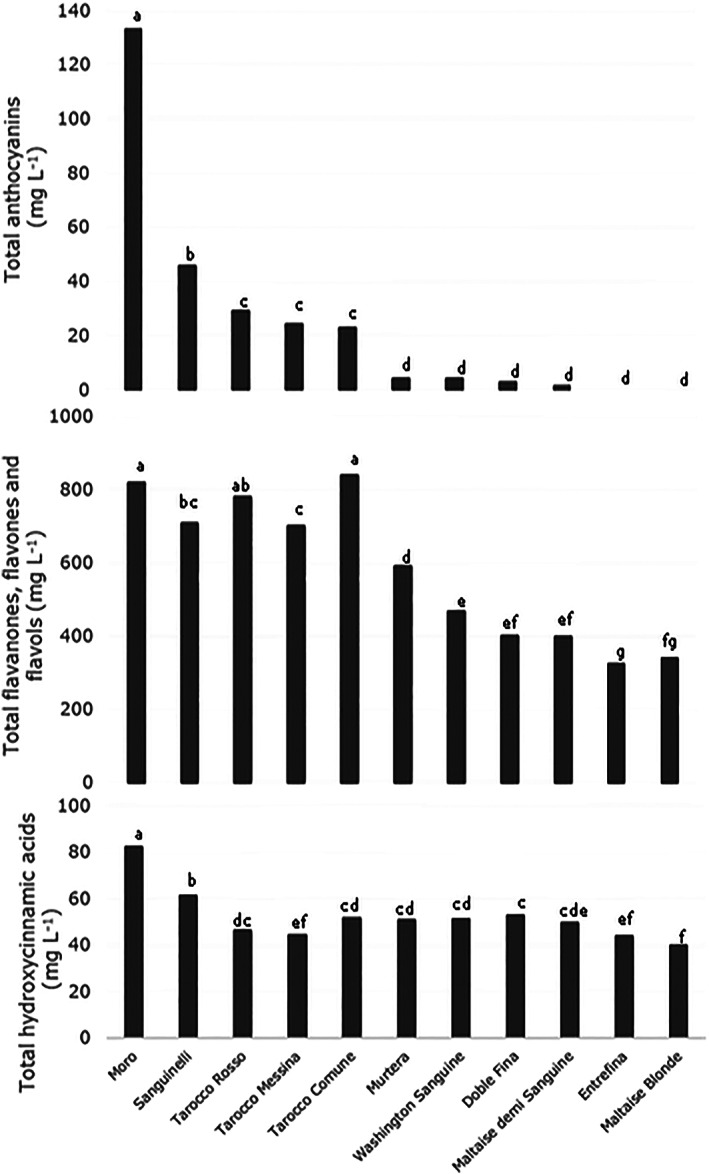
Content of anthocyanins, flavanones and flavones, and hydroxycinnamic acids of the eleven blood orange juice. Data are the mean ± SD (*n* = 6). Values followed by the same letter, within the same row, are not significant different according to Fisherʼs LSD procedure at 95% confidence level.

Total anthocyanin content appeared in Fig. 2. The highest level of total anthocyanins was in Moro (133.10 mg L^−1^). Next was Sanguinelli, which had less than half of this amount (45.59 mg L^−1^). No significant differences were observed between the Tarocco cultivars.

Flavanones, flavones and flavonols varied significantly from 325.97 mg L^−1^ to 841.63 mg L^−1^. Entrefina was lowest in this matrix of chemical markers, followed by Maltaise Blonde, Maltaise demi Sanguine and Doble Fina, for which the values were all below 400 mg L^−1^. The highest values, greater than 800 mg L^−1^, were found in Tarocco Comune, followed by Moro. Considering the hydroxycinnamic acids, the highest level was again in Moro (82.43 mg L^−1^), whereas the lowest was in Maltaise Blonde (39.96 mg L^−1^).

### Individual phenolic compounds

The different subclasses of phenolic compounds found in blood oranges juices are listed in Table 5. We identified eight phenolic acids, with *p*‐coumaric acid as the main compound in all cultivars; Moro (35.82 mg L^−1^), and Sanguinelli (27.95 mg L^−1^) were the juices that were richer in this compound. Concerning ferulic acid, the highest content was found again in Moro and Sanguinelli. Another major phenolic component was *p*‐coumaroylquinic acid found in Moro (5.26 mg L^−1^), Tarocco Rosso (4.06 mg L^−1^), Maltaise Blonde (4.24 mg L^−1^) and Tarocco Comune (3.94 mg L^−1^). The content of *p*‐coumaroylquinic acid was lowest in Tarocco Messina (1.71 mg L^−1^).

**Table 5:** Content (mg L^−1^ of juice) of individual phenolic subclasses in blood orange juice

Compounds	Peak no.	Rt (min)	MW	Doble Fina	Entrefina	Maltaise Blonde	Maltaise demi Sanguine	Moro	Murtera	Sanguinelli	Tarocco Comune	Tarocco Messina	Tarocco Rosso	Washington Sanguine
Hydroxycinnamic acids														
Feruloyl hexose	1	13.6	356	2.5 ± 0.04 e	4.68 ± 0.07 b	3.59 ± 0.02 c	4.88 ± 0.03 b	5.08 ± 0.21 b	2.85 ± 0.02 de	3.58 ± 0.13 c	4.92 ± 0.08 b	4.69 ± 0.01 b	6.11 ± 0.36 a	3.02 ± 0.06 d
Feruloyl quinic acid 1	2	14.7	368	2.65 ± 0.07 d	1.23 ± 0.05 e	1.61 ± 0.08 e	2.5 ± 0.07 d	4.99 ± 0.02 a	3.19 ± 0.03 cd	2.77 ± 0.01 d	3.58 ± 0.46 bc	3.02 ± 0.6 cd	4.14 ± 0.4 b	2.69 ± 0.05 d
P‐coumaroyl quinic acid	3	16.0	338	3.46 ± 0.74 bc	3.14 ± 0.24 bcd	4.24 ± 1.33 ab	2.27 ± 0.18 cd	5.26 ± 0.25 a	2.25 ± 0.02 cd	3.26 ± 0.15 bc	3.94 ± 0.25 ab	1.71 ± 0.41 d	4.06 ± 0.18 ab	2.32 ± 0.18 cd
Feruloyl quinic acid 2	4	16.3	368	7.58 ± 1.06 cd	5.41 ± 0.74 e	6.99 ± 0.25 cde	9.56 ± 0.15 ab	8.52 ± 0.44 abc	8.02 ± 0.08 bcd	6.86 ± 0.16 de	9.89 ± 0.28 a	7.81 ± 0.86 cd	9.74 ± 0.61 a	7.32 ± 0.3 cd
Feruloyl quinic acid 3	5	17.9	368	3.24 ± 0.02 b	6.33 ± 0.5 a	2.42 ± 0.88 bc	1.16 ± 0.04 c	7.27 ± 0.22 a	1.88 ± 0.03 c	2.46 ± 0.17 bc	3.35 ± 1 b	1.64 ± 0.14 c	1.37 ± 0.14 c	1.92 ± 0.04 c
Sinapic acid	6	18.5	224	5.6 ± 0.25 cd	7.35 ± 0.46 b	4.62 ± 0.37 d	6.98 ± 0.01 bc	10.3 ± 1 a	4.72 ± 0.07 d	8.05 ± 0.35 b	7.23 ± 0.24 b	4.71 ± 1.09 d	7.72 ± 0.51 b	5.3 ± 0.07 d
*p*‐Coumaric acid	7	21.2	164	23.18 ± 1.05 c	14.78 ± 0.11 ef	14.43 ± 0.27 f	18.41 ± 0.27 d	35.82 ± 2.42 a	22.97 ± 0.14 c	27.95 ± 0.93 b	16.29 ± 1.42 def	17.84 ± 0.23 de	13.2 ± 0.98 f	25.31 ± 1.07 bc
Ferulic acid	8	21.4	194	7.01 ± 0.11 def	5.75 ± 0.13 ef	5.64 ± 0.26 f	8.65 ± 0.41 bc	10.25 ± 0.87 a	7.78 ± 0.21 cd	10.03 ± 0.37 ab	7.17 ± 0.19 de	7.59 ± 1.11 cd	6.09 ± 0.43 ef	6.53 ± 0.11 def
Flavanones, flavones and flavonols														
Vicenin 2	9	22.1	594	114.24 ± 2.47 b	66.17 ± 1.02 e	83.68 ± 1.27 c	72.68 ± 0.65 ed	120.38 ± 2.07 ab	116.8 ± 1.67 b	129.5 ± 0.75 a	117.32 ± 1.55 b	117.22 ± 10.72 b	80.63 ± 2.86 cd	128.62 ± 0.87 a
Isorhamnetin‐3‐*O*‐rutinoside	10	31.0	624	2.37 ± 0.01 c	1.52 ± 0.04 d	1.61 ± 0.15 d	1.8 ± 0.16 d	3.12 ± 0.17 b	3.28 ± 0.04 b	2.74 ± 0.01 c	1.78 ± 0.24 d	3.75 ± 0.14 a	3.26 ± 0.11 b	2.5 ± 0.04 c
Naringin	11	30.2	580	43.8 ± 1.52 h	57.64 ± 1.51 fg	36.44 ± 0.19 h	55.85 ± 0.79 g	196.1 ± 2.36 a	85.89 ± 1.4 c	107.64 ± 5.09 b	85.46 ± 1.12 c	78.51 ± 4.68 cd	71.66 ± 6.21 ed	65.7 ± 1.85 ef
Rutin	12	27.2	610	4.07 ± 0.06 cd	2.28 ± 0.04 f	2.86 ± 0.01 e	2.77 ± 0.07 e	7.3 ± 0.1 a	4.24 ± 0.05 c	5.02 ± 0.12 b	3.89 ± 0.19 cd	3.92 ± 0.18 cd	4.76 ± 0.26 b	3.82 ± 0.02 d
Hesperidin	13	32.0	610	238.22 ± 8.02 d	198.34 ± 9.7 d	214.86 ± 2.15 d	265.05 ± 24.11 d	491.83 ± 62.1 b	380.18 ± 23.94 c	465.54 ± 11.26 b	633.17 ± 16.78 a	499.31 ± 8.12 b	621.67 ± 6.91 a	266.75 ± 23.9 d
Anthocyanins														
Cyanidin‐3‐*O*‐glucoside	14	18.7	449	1.18 ± 0.1 d	0 ± 0 d	0 ± 0 d	0.49 ± 0.08 d	69.43 ± 2.17 a	1.41 ± 0.06 d	20.95 ± 0.99 b	8.68 ± 0.22 c	8.28 ± 1.48 c	10.21 ± 1.68 c	1.8 ± 0.05 d
Cyanidin‐3‐(6′′‐malonyl)‐glucoside	15	22.7	535	1.65 ± 0.11 e	0 ± 0 e	0 ± 0 e	0.88 ± 0.13 e	63.47 ± 1.61 a	2.75 ± 0.06 e	24.63 ± 1.54 b	14.24 ± 0.01 d	16.11 ± 1.81 cd	18.84 ± 2.35 c	2.26 ± 0.01 e

Data are the mean ± SD (*n* = 6). Values followed by the lowercase same letter, within the same column, are not significant different according to Fisherʼs LSD procedure at 95% confidence level.

Among the flavonoids found in the 11 cultivars examined, the predominant one was hesperidin, a flavanone glycoside. In our results, the highest values, greater than 500 mg L^−1^, were found in Taroccoʼs clones Comune, followed by Moro and Sanguinelli. Entrefina, Maltaise Blonde, Maltaise demi Sanguine, Washington Sanguine and Doble Fina showed moderate values of hesperidin, in the range between 198 and 266 mg L^−1^. Naringin, another flavanone glycoside, showed the lowest content in Maltaise Blonde and Doble Fina, and the highest in Moro and Sanguinelli.

Among all cultivars, the lowest juice concentrations of anthocyanins was found in Doble Fina, whereas Moro, which had the deepest red juice colour, had the highest contents of cyanidin‐3‐*O*‐glucoside and cyanidin‐3‐(6′′‐malonyl)‐glucoside.

**Figure 3:**
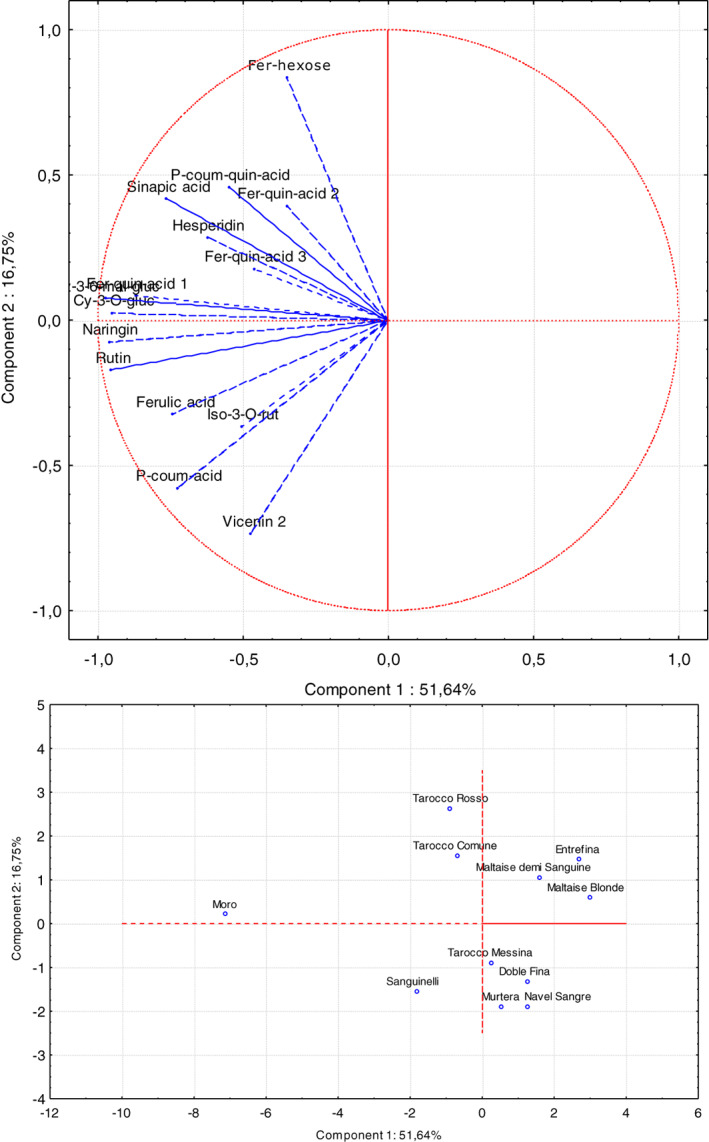
Principal component analysis of the individual phenolics (A) in blood orange juice of the eleven cultivars (B) (average of 2 years). Data set: eleven cultivars (Doble Fina, Entrefina, Maltaise Blonde, Maltaise demi Sanguine, Moro, Murtera, Sanguinelli, Tarocco Comune, Tarocco Messina, Tarocco Rosso, Washington Sanguine); fifteen phenolic compounds (hydroxycinnamic acids: feruloyl hexose, feruloyl quinic acid 1, p‐coumaroyl quinic acid, feruloyl quinic acid 2, feruloyl quinic acid 3, sinapic acid, p‐coumaric acid, ferulic acid. flavanones, flavones and flavonols: vicenin 2, isorhamnetin‐3‐O‐rutinoside, naringin, rutin, hesperidin. anthocyanins: cyanidin‐3‐O‐glucoside, cyanidin‐3‐(6′′‐malonyl)‐glucoside).

The 2‐year mean of the entire set of parameters measured during the experiment was evaluated via PCA. The first two principal components accounted for 68.39% of the total explained variance. PC1 (51.64% of variance explained) was highly correlated with cyanidin‐3‐*O*‐glucoside, cyanidin‐3‐(6′′‐malonyl)‐glucoside, naringin and rutin (Fig. 3). PC2 (16.75% of variance explained) was positively correlated mostly with some hydroxycinnamic acids (ferulolyl hexose), whereas *p*‐coumaric acid and ferulic acid were negatively correlated with the genotypes.

Considering the first two principal components, Moro was the cultivar that showed far the highest negative score (−7.2), correlated with anthocyanins, naringin and rutin, followed by Sanguinelli, Tarocco Rosso and Tarocco Comune (−2.0, −1.2 and − 1.0, respectively). Conversely, Maltaise blonde and Entrefina showed the highest scores (3.0 and 2.8, respectively).

## DISCUSSION

### Physicochemical properties

Considering fruit weight parameters, Tarocco clones were confirmed to be among those with higher values,^21^ with the exception of the old line Tarocco Comune, which indeed is no longer cultivated in modern citriculture. Conversely, Sanguinelli is characterized by the small size of the fruit, which limits its use for the fresh market, instead being preferred for processing.^22^ Some of the cultivars were more or less isodiametric (e.g. Maltaise demi Sanguine, Tarocco Comune, Murtera and Entrefina) and others were more elongated. Moro had the thickest rind and, indeed, it is preferred for transport to distant markets for its greater resistance to mechanical damage.^21^ Tarocco Comune and Tarocco Messina were those with the thinnest rind: this aligns with the easy peelability of the Tarocco cultivars,^23^ a usual feature of mandarin fruits.^24^

The values of TSS and TA are the most relevant determinants of juice quality. We observed significant differences between the cultivars evaluated. Fruits of Tarocco Comune showed the highest TSS content (15.03 °Brix), whereas Tarocco Messina fruits, usually considered a late‐maturing cultivar, recorded the lowest TSS (11.33 °Brix). The TSS values of both blonde and red orange cultivars are usually reported to range between 11 and 15 °Brix, depending on maturity, environmental conditions and agronomical practices.^20^, ^25^ All of the pigmented cultivars registered values of total acidity of between 11 and 14 g citric acid L^−1^, and the characteristic taste of the juice show a high acidity, markedly different from the blonde orange varieties. At physiological maturity, the latter normally reach values well below 10 g citric acid L^−1^,^20^, ^26^ thus ensuring sweetness.

This is confirmed by the TSS/TA ratio, an important indicator of commercial and sensory ripeness. This is used widely for citrus fruits because it helps define their characteristic flavour. The European Commission determined the maturity requirements for orange fruits and, among these requirements, the minimum sugar/acid ratio for commercialisation is 6.5, regardless of cultivar or fruit type (blood or navel orange).^27^ Here, the maturity indices of all the pigmented cultivars fell within the range 8.7 to 11.3, but did not achieve the higher values recorded in some blonde oranges.^20^, ^26^ This is because, during ripening, the acids decrease more slowly in the blood oranges than in the blonde ones.^23^

### External rind and juice colour

The activation of the anthocyanin biosynthetic pathway in the rind and flesh of blood oranges is one of the major differences distinguishing these cultivars from blonde ones.^28^ The deepness of the red colour is strongly attractive to consumers. Fruit pigmentation is also increasingly appreciated for its high nutraceutical value as a result of the presence of bioactive compounds.^29^, ^30^ Peel and flesh colour are not linked, and so strong peel colour did not necessarily correspond to strong flesh colour, confirming the previous results.^13^ Evaluating *H*° and CCI values, the cultivars that showed the deepest red colour were Sanguinelli for the rind and Moro for the juice, in accordance with previous research.^10^, ^13^, ^31^

Generally, observing all the colour parameters evaluated (*L*, a*, b*, C*, *H*°, CCI), the CCI appears to be most useful for distinguishing between these cultivars, more so than *H*° that is commonly used for colour characterisation and considered an objective and reliable parameter to evaluate the maturity indices of citrus fruits.^23^, ^32^ Also, the external colour in red oranges has been used widely as a good indicator of fruit maturity. Examining the values of both the peel and juice colours, it is evident that the intensity of the red colour is different because the anthocyanin biosynthesis pathways are not the same.^32^, ^33^, ^34^ Furthermore, in the Tarocco clones, there is a frequent lack of correspondence between the intensities of the peel and flesh pigmentations.^23^

### Organic acids and sugars

The accumulation of soluble sugars and the decline in acid content (mainly citric acid) in blood oranges are typical changes taking place in the pulp during maturation of citrus fruits. The sweetness of an orange depends on the sugars, which are the major components of the juice. The organic acid composition is also important because it influences the sensory properties of the juice.^35^ Blood oranges are generally more acid than blonde ones at harvest.^23^, ^25^ The main organic acid found in the oranges under investigation was citric acid, followed by malic acid and ascorbic acids. Juices from Moro and Sanguinelli showed the highest acidic content, as broadly reported.^22^, ^31^

The quality of a citrus fruit is expressed through the levels of a range of chemical compounds including vitamins, mineral elements and phenolics that have important antioxidant activity.^36^ Among the vitamins, vitamin C is calculated as ascorbic acid and it is present in high amounts in the fresh juice.^13^ Blood orange is a rich source of vitamin C; indeed, levels in Moro and Tarocco have higher concentrations than many blonde orange varieties, ranging from 0.50 to 0.80 g kg^−1^ of juice.^37^ In our results, values ranged from 0.35 to 0.50 g kg^−1^, being quite similar to that reported in previous research.^22^

As mentioned, the sweetness of orange juice depends on the levels of the common sugars: sucrose, glucose and fructose. Taken together, these account for about 80% of the total soluble solids in orange juice and the ratio sucrose:glucose:fructose is generally approximately 2:1:1.^38^ The glucose:fructose ratio is a key indicator for determining the authenticity of citrus juices and it is generally higher than 0.85. In most other fruits, the level of glucose exceeds that of fructose but oranges contain glucose and fructose in almost equal quantities and, sometimes, fructose can be slightly more concentrated than glucose as also found in our study.^39^ Our results were similar to, and consistent with, those reported in other studies.^40^

### Polyphenols and total antioxidant activity

Citrus fruits are rich in phenolic acids and flavonoids, two major groups of natural antioxidants on which most of their functional properties are based. The variability in flavonoid composition among fruits is mainly attributable to genotype.^41^ These secondary metabolites participate in various functions in the plant; particularly, in the fruit, they are associated with colour, sensory characteristics (flavour, astringency, hardness), nutritional characteristics and antioxidant activity.^42^ In the present study, the total phenolic content ranged between 117.26 and 241.91 mg of gallic acid 100 g^−1^. Even if Tarocco clones were the cultivars with the highest TPC content, our results were generally lower than those reported in previous studies.^43^, ^44^

Antioxidant activity of different foods has been thoroughly investigated because of their counteracting oxidation processes that prevent the chronic diseases related to oxidative stress in human body.^14^ Different types of antioxidant compounds, such as ascorbic acid, flavonoids and phenolic acids, were considered to be natural sources in horticultural products. For this reason, the ABTS assay was used to evaluate the antioxidant capacity of both lipophilic and hydrophilic antioxidants, including flavonoids (flavones, flavanones and flavonols) and phenolic acids (among all, ferulic acid and *p*‐coumaric acid).^43^ Previous research on blood oranges revealed that the higher amounts of anthocyanins play an important role in the antioxidant activity of the juice.^22^, ^45^

Hydrophilic antioxidant activity was far more prevalent than lipophilic antioxidant activity, as reported in other research.^18^, ^46^ In accordance with previous results,^47^ Moro showed the highest level, followed by Sanguinelli; by contrast, other studies^40^ reported higher levels in Taroccoʼs clones than in Moro.

### Total anthocyanins, total hydroxycinnamic acids and phenolic composition

Anthocyanins synthetized and accumulated in blood oranges impart their distinctive purple–red colouration to both the peel and juice.^2^, ^31^ In line with other studies,^43^, ^48^ we found the highest level of total anthocyanins in Moro, followed by Sanguinelli. No significant differences were observed between the Tarocco cultivars, whereas lower levels were found in the remaining cultivars. The different levels of these pigments recorded in the present study confirm that their accumulation process is genetically driven, rather than environmental, because all the abiotic conditions (the aerial and soil microenvironments) were identical in our collection area.

Oranges and other citrus fruits have high concentrations of phenolics and flavonoids, mainly linked to the flavanones group.^49^ The total phenolic composition in citrus fruit may range from approximately 300 to 1200 mg L^−1^, and depends on the species, growing season, ripening and environmental factors.^39^

In the present study, the total phenolic compound ranged between 326 and 842 and the highest content of total flavanones, flavones and flavonols was in Moro, followed by Tarocco comune, as reported in previous studies.^37^, ^47^

Previous studies^50^ showed that hydroxycinnamic acids were distinct for the blood orange cultivars, being much more abundant in the blood than in the blonde orange juices. We found Moro had the greatest amount of total hydroxycinnamic acids, which falls in line with several previous studies,^31^, ^48^ with decreasing concentrations in Sanguinelli.

### Individual phenolic compounds

The citrus species contain multiple classes of polyphenols, each possessing a spectrum of chemical behaviours and bioactivities. The hydroxycinnamic acids represent an important group of compounds that derive from the general phenylpropanoid pathway.^31^ In the present study, eight phenolic acids were assessed, and *p*‐coumaric acid was the main hydroxycinnamic acid identified in all cultivars, followed by ferulic and sinapic acid. These data are in contrast to what reported in literature,^47^, ^50^ where ferulic or chlorogenic^31^ acids were found to be the most dominant hydroxycinnamic acid in some blood orange cultivars. In the present study, the highest content of ferulic acid was found in Moro and Sanguinelli. *p*‐Coumaroylquinic acid was found in high amounts in Moro and Taroccoʼs clones, with the exception of Tarocco Messina. This may have been because the latter is a very late‐maturing cultivar.

The presence of naringin and hesperidin in blood oranges has been reported in previous studies.^31^, ^47^ The predominant flavonoid found in the 11 cultivars evaluated in the present study was hesperidin, in line with previous research.^13^, ^51^, ^52^ In a previous investigation on blood oranges,^53^ Sanguinello and Tarocco presented a similar profile to the one obtained in the present study, in which Tarocco showed greater amount than Moro. Naringin is confirmed to be present in a higher amount in Moro with respect to Tarocco cultivars.^31^ Anthocyanin content, as noted, is related to internal fruit colour. As previously reported,^43^ cyanidin 3‐glucoside and cyanidin 3‐(6′′‐malonylglucoside) were predominant in blood orange, especially in Moro. We found the lowest concentrations of anthocyanins in juices from Doble Fina, Entrefina, Maltaise Blonde, Maltaise demi Sanguine, Murtera and Washington Sanguine, whereas Moro, as expected, had the highest contents of cyanidin‐3‐*O*‐glucoside and cyanidin‐3‐(6′′‐malonyl)‐glucoside. The content of all these phenolic compounds is known to depend strongly on both species and cultivar.^54^, ^55^, ^56^

Previous studies^46,47^ reported a positive trend for the increase of most bioactive compounds in blood oranges, such as organic acids (ascorbic acid, malic acid and citric acid), phenolic compounds (didymin, narirutin, and vicenin‐2), anthocyanin compounds (cyanidin 3‐*O*‐glucoside and cyanidin 3‐*O*‐(6′′‐acetyl) glucoside) and antioxidant activity.

The PCA analysis permitted to highlight the influence of the genotype on the biosynthesis of the anthocyanins and of the other phenolics. Moro and Sanguinelli were the varieties with the highest content of phenols, and the results of the present study indicate the strong influence of the cultivars on the classes of polyphenols investigated.

## CONCLUSIONS

Blood oranges are an excellent source of natural antioxidants and bioactive compounds such as phenols, flavonoids, anthocyanins and ascorbic acid. In Spain, their cultivation represents only a relatively small part of total citrus production. Nevertheless, consumer interest in blood oranges is increasing because of their perceived valuable nutraceutical properties. Hence, a nutraceutical characterisation of the main blood orange cultivars is timely, especially one that compares the autochthonous Spanish cultivars with several others that are more widespread elsewhere in the Mediterranean basin. In the present study, we analysed the colour of both the peel and the juice, making comparisons using different colour index parameters. Moro was the darkest red orange cultivar, followed by Sanguinelli. Maturity index was another important parameter related to the qualitative characteristics of citrus fruit and is linked to fruit ripeness. Tarocco Rosso had the highest value as a result of its low citric acid content. The results show that the juice of Moro has the greatest levels of total flavonoids, total phenolics and total anthocyanins, followed by Sanguinelli.

## References

[1] Food and Agriculture Organization of the United Nations . FAOSTAT Statistical Database. [Rome]. Available: www.faostat.fao.org

[2] Lo Piero AR , The state of the art in biosynthesis of anthocyanins and its regulation in pigmented sweet orange [(*Citrus sinensis*) L, Osbeck]. J Agric Food Chem 163:4031–4041 (2015). 10.1021/acs.jafc.5b01123 25871434

[3] Lana G , Modica G , Las Casas G , Siracusa L , La Malfa S , Gentile A *et al*., Molecular insights into the effects of rootstocks on maturation of blood oranges. Horticulturae 7:468 (2021).

[4] Pannitteri C , Continella A , Lo Cicero L , Gentile A , La Malfa S , Sperlinga E *et al*., Influence of postharvest treatments on qualitative and chemical parameters of tarocco blood orange fruits to be used for fresh chilled juice. Food Chem 230:441–447 (2017). 28407933 10.1016/j.foodchem.2017.03.041

[5] Habibi F , Ramezanian A , Guillén F , Castillo S , Serrano M and Valero D , Changes in bioactive compounds, antioxidant activity, and nutritional quality of blood orange cultivars at different storage temperatures. Antioxidants 9:1016 (2020). 10.3390/antiox9101016 PMC7589990 33092024

[6] Continella A , Pannitteri C , La Malfa S , Legua P , Distefano G , Nicolosi E *et al*., Influence of different rootstock on yield precocity and fruit quality of “Tarocco Sciré” pigmented sweet orange. Sci Hortic 230:62–67 (2018).

[7] Grosso G , Galvano F , Mistretta A , Marventano S , Nolfo F , Calabrese G *et al*., Red orange: experimental models and epidemiological evidence of its benefits on human health. Oxid Med Cell Longev 11:1–11 (2013). 10.1155/2013/157240 PMC3659473 23738032

[8] Proteggente AR , Saija A , De Pasquale A and Rice Evans C , The compositional characterisation and antioxidant activity of fresh juices from sicilian sweet orange (*Citrus sinensis* L. Osbeck) varieties. Free Radic Res 37:681–687 (2003). 12868495 10.1080/1071576031000083198

[9] Ministerio de Agricultura, Pesca y Alimentacion, Anuario de estadistica 2019. Available: https://www.mapa.gob.es/es/estadistica/temas/publicaciones/anuario-de-estadistica/2019/

[10] Porras IC , Conesa Martínez A , Martínez Nicolás J , Jara Rodríguez FJ , Manera Bassa F , Medina Sánchez‐Valladares A *et al*., Estudio preliminar de diversas variedades de naranjas sanguinas. Parte i: parámetros de calidad y del colour externo de los frutos. Levane Agricola 420:19–22 (2014).

[11] Saunt J , Citrus Varieties of the World, 2nd edn. Sinclair International Limited, Norwich, UK (2000).

[12] Rapisarda P , Bianco ML , Pannuzzo P and Timpanaro N , Effect of cold storage on vitamin C, phenolics and antioxidant activity of five orange genotypes [*Citrus sinensis* (L.) Osbeck]. Postharvest Biol Technol 49:348–354 (2009).

[13] Cebadera‐Miranda L , Domínguez L , Dias MI , Barros L , Ferreira ICFR , Igual M *et al*., Sanguinello and Tarocco (*Citrus sinensis* [L.] Osbeck): bioactive compounds and colour appearance of blood oranges. Food Chem 270:395–402 (2019). 30174063 10.1016/j.foodchem.2018.07.094

[14] Cömert DE and Gökmen V , Evolution of food antioxidants as a core topic of food science for a century. Food Res Int 105:76–93 (2018). 29433271 10.1016/j.foodres.2017.10.056

[15] Wang LS and Stoner GD , Anthocyanins and their role in cancer prevention. Cancer Lett 269:281–290 (2008). 18571839 10.1016/j.canlet.2008.05.020 PMC2582525

[16] McGuire RG , Reporting of objective color measurements. HortScience 27:1254–1255 (1992).

[17] Jimenez‐Cuesta M , Cuquerella J , Martínez Javega JM , Determination of a colour index for citrus fruit degreening, in *Proceedings of the International Society of Citriculture Citriculture, IV Congress,Tokio, Japan*, Vol. 2, pp. 750–753. (1981).

[18] Legua P , Forner J , Hernández F and Forner‐Giner M , Physicochemical properties of orange juice from ten rootstocks using multivariate analysis. Sci Hortic 160:268–273 (2013).

[19] Singleton VL , Orthofer R and Lamuela‐Raventos RM , [14] Analysis of total phenols and other oxidation substrates and antioxidants by means of folin‐ciocalteu reagent. Methods Enzymol 299:152–178 (1999).

[20] Legua P , Bellver R , Forner J and Forner‐Giner MA , Plant growth, yield and fruit quality of ‘Lane Late’ navel orange on four citrus rootstocks. Span J Agric Res 9:271–279 (2011).

[21] Caruso M , Ferlito F , Licciardello C , Allegra M , Strano MC , Di Silvestro S *et al*., Pomological diversity of the Italian blood orange germplasm. Sci Hortic 213:331–339 (2016).

[22] Ordóñez‐Díaz JL , Hervalejo A , Pereira‐Caro G , Muñoz‐Redondo JM , Romero‐Rodríguez E , Arenas‐Arenas FJ *et al*., Effect of rootstock and harvesting period on the bioactive compounds and antioxidant activity of two Orange cultivars (‘Salustiana’ and ‘Sanguinelli’) widely used in juice industry. Processes 8:1212 (2020).

[23] Rapisarda P , and Russo G , Fruit quality of five Tarocco selections grown in Italy, in *Proceedings of the International Society of Citriculture. 9th Congress*. Univ. of California, Riverside, CA, Vol. 2, pp. 1149–1153 (2000).

[24] Caruso M , Continella A , Modica G , Pannitteri C , Russo R , Salonia F *et al*., Rootstocks influence yield precocity, productivity, and pre‐harvest fruit drop of mandared pigmented mandarin. Agronomy 10:1305 (2020).

[25] Lado J , Gambetta G and Zacarias L , Key determinants of citrus fruit quality: metabolites and main changes during maturation. Sci Hortic 233:238–248 (2018).

[26] Hervalejo A , Cardeñosa V , Forner‐Giner MA , Salguero A , Pradas IC , Moreno JM *et al*., Preliminary data on influence of six citrus rootstocks on fruit quality of 'Lane Late' navel orange. Acta Hort 1065:363–366 (2015).

[27] Anon . Commission Implementing Regulation (EC) N° 543/2011 of 7 June 2011 laying down detailed rules for the application of the Council Regulation (EC) No 1243/2007 in respect of the fruit and vegetables processed fruit and vegetables sectors. Official Journal of the European Union, L 157, 15 June (2011).

[28] Butelli E , Licciardello C , Zhang Y , Liu J , Mackay S , Bailey P *et al*., Retrotransposons control fruitspecific, cold‐dependent accumulation of anthocyanins in blood oranges. Plant Cell 24:1242–1255 (2012). 22427337 10.1105/tpc.111.095232 PMC3336134

[29] Dugo P , Mondello L , Morabito D and Dugo G , Characterization of anthocyanin fraction of Sicilian blood orange juice by Micro‐HPLC‐ESI/MS. J Agric Food Chem 51:1173–1176 (2003). 12590452 10.1021/jf026078b

[30] De Pascual TS and Sanchez‐Ballesta MT , Anthocyanins: from plant to health. Phytochemistry 7:281–299 (2008).

[31] Morales J , Bermejo A , Navarro P , Forner‐Giner MA and Salvador A , Rootstock effect on fruit quality, anthocyanins, sugars, hydroxycinnamic acids and flavanones content during the harvest of blood oranges ‘Moro’ and ‘Tarocco Rosso’ grown in Spain. Food Chem 342:128305 (2021). 33097323 10.1016/j.foodchem.2020.128305

[32] Lado J , Rodrigo MJ and Zacarías L , Maturity indicators and citrus fruit quality. Stewart Postharvest Rev 10:2 (2014).

[33] Chaves‐Silvaab S , Luís dos Santos A , Chalfun‐Júnior A , Zhao J , Peres LEP and Benedito VA , Understanding the genetic regulation of anthocyanin biosynthesis in plants. Tools for breeding purple varieties of fruits and vegetables. Phytochemistry 153:11–27 (2018). 29803860 10.1016/j.phytochem.2018.05.013

[34] Lo Cicero L , Puglisi I , Nicolosi E , Gentile A , Ferlito F , Continella A *et al*., Anthocyanin levels and expression analysis of biosynthesis‐related genes during ripening of sicilian and international grape berries subjected to leaf removal and water deficit. J Agric Sci Technol 18:1333–1344 (2016).

[35] Legua P , Forner JB , Hernàndez F and Forner‐Giner MA , Total phenolics, organic acids, sugars and antioxidant activity of mandarin (*Citrus clementina* Hort. ex Tan.): variation from rootstock. Sci Hortic 174:60–64 (2014).

[36] Zhuo Z , Xi W , Hu Y , Nie C and Zhou Z , Antioxidant activity of citrus fruits. Food Chem 196:885–896 (2016). 26593569 10.1016/j.foodchem.2015.09.072

[37] Rapisarda P , Tomaino A , Lo Cascio R , Bonina F , De Pasquale A and Saija A , Antioxidant effectiveness as influenced by phenolic content of fresh orange juices. J Agric Food Chem 47:4718–4723 (1999). 10552879 10.1021/jf990111l

[38] Lee HS and Coates GA , Quantitative study of free sugars and myoinositol in citrus juices by HPLC and literature compilation. J Liq Chromatogr Relat Technol 14:2123–2141 (2000).

[39] Kelebek H and Selli S , Determination of volatile, phenolic, organic acid and sugar components in a Turkish cv. Dortyol (*Citrus sinensis* L. Osbeck) orange juice. J Sci Food Agric 91:1855–1862 (2011). 21480267 10.1002/jsfa.4396

[40] Sicari V , Pellicanò TM , Giuffrè AM , Zappia C and Capocasale M , Bioactive compounds and antioxidant activity of citrus juices produced from varieties cultivated in Calabria. J Food Meas Charact 10:773–780 (2016).

[41] Hunlun C , De Beer D , Sigge GO and Van Wyk J , Phenolic composition and total antioxidant capacity of South African frozen concentrated orange juice as affected by varietal, seasonal and regional differences. J Sci Food Agric 99:1029–1037 (2019). 30009498 10.1002/jsfa.9267

[42] Simón‐Grao S , Gimeno V , Simón I , Lidón V , Nieves M , Balal RM *et al*., Fruit quality characterization of eleven commercial mandarin cultivars in Spain. Sci Hort 165:274–280 (2014).

[43] Rapisarda P , Fabroni S , Peterek S , Russo G and Mock HP , Juice of new citrus hybrids (*Citrus clementina* Hort. ex Tan *C. Sinensis* L. Osbeck) as a source of natural antioxidants. Food Chem 117:212–218 (2009).

[44] Letaief H , Zemni H , Mliki A and Chebil S , Composition of Citrus sinensis (L.) Osbeck cv «Maltaise demi‐sanguine» juice. A comparison between organic and conventional farming. Food Chem 194:290–295 (2016). 26471557 10.1016/j.foodchem.2015.08.025

[45] Modica G , Pannitteri C , Di Guardo M , La Malfa S , Gentile A , Ruberto G *et al*., Influence of rootstock genotype on individual metabolic responses and antioxidant potential of blood orange cv. Tarocco Scirè. J Food Compos Anal 105:104246 (2021).

[46] Reche J , Almansa MS , Hernández F , Amorós A and Legua P , Physicochemical and antioxidant capacity of jujube (Ziziphus jujuba Mill.) at different maturation stages. Agronomy 11:132 (2021).

[47] Kelebek H , Canbas A and Selli S , Determination of phenolic composition and antioxidant capacity of blood orange juices obtained from cvs. Moro and Sanguinello (*Citrus sinensis* (L.) Osbeck) grown in Turkey. Food Chem 107:1710–1716 (2008).

[48] Fallico B , Ballistreri G , Arena E , Brghina S and Rapisarda P , Bioactive compounds in blood oranges (*Citrus sinensis* (L.) Osbeck): level and intake. Food Chem 215:67–75 (2017). 27542451 10.1016/j.foodchem.2016.07.142

[49] Pereira‐Caro G , Borges G , van der Hooft J , Clifford MN , Del Rio D , Lean ME *et al*., Orange juice (poly)phenols are highly bioavailable in humans. Am J Clin Nutr 100:1378–1384 (2014). 25332336 10.3945/ajcn.114.090282

[50] Rapisarda P , Carollo G , Fallico B , Tomaselli F and Maccarone E , Hydroxycinnamic acids as markers of Italian blood orange juices. J Agric Food Chem 46:464–470 (1998). 10554264 10.1021/jf9603700

[51] Gattuso G , Barreca D , Gargiulli C , Leuzzi U and Caristi C , Flavonoid composition of citrus juices. Molecules 12:1641–1673 (2007). 17960080 10.3390/12081641 PMC6149096

[52] Barreca D , Bellocco E , Leuzzi U and Gattuso G , First evidence of C‐ and Oglycosyl flavone in blood orange (*Citrus sinensis* (L.) Osbeck) juice and their influence on antioxidant properties. Food Chem 149:244–252 (2014). 24295703 10.1016/j.foodchem.2013.10.096

[53] Barreca D , Gattuso G , Laganà G , Leuzzi U and Bellocco E , C‐ and O‐glycosyl flavonoids in Sanguinello and Tarocco blood orange (*Citrus sinensis* (L.) Osbeck) juice: identification and influence on antioxidant properties and acetylcholinesterase activity. Food Chem 196:619–627 (2016). 26593535 10.1016/j.foodchem.2015.09.098

[54] Arena E , Fallico B and Maccarrone E , Evaluation of antioxidant capacity of blood orange juices as influenced by constituents, concentration process and storage. Food Chem 74:423–427 (2001).

[55] Ballistreri G , Continella A , Gentile A , Amenta M , Fabroni S and Rapisarda P , Fruit quality and bioactive compounds relevant to human health of sweet cherry (*Prunus avium* L.) cultivars grown in Italy. Food Chem 140:630–638 (2013). 23692746 10.1016/j.foodchem.2012.11.024

[56] Todaro A , Cavallaro R , La Malfa S , Continella A , Gentile A , Fischer UA *et al*., Anthocyanin profile and antioxidant activity of freshly squeezed pomegranate (*Punica granatum* L.) juices of Sicilian and Spanish provenances. *It* . J Food Sci 28:464–479 (2016).

